# A Case of Stoma Limb Perforation Nine Years After Abdominoperineal Resection

**DOI:** 10.7759/cureus.81347

**Published:** 2025-03-28

**Authors:** Hideo Kidogawa, Junya Noguchi, Masao Inoue, Satoshi Kimura, Kohji Okamoto

**Affiliations:** 1 Department of Surgery, Kitakyushu City Yahata Hospital, Kitakyushu, JPN; 2 Department of Clinical Pathology, Kitakyushu City Yahata Hospital, Kitakyushu, JPN

**Keywords:** abdominoperineal resection, colonic diverticulum perforation, diverticulitis, sigmoid colostomy, stoma limb perforation

## Abstract

Stoma limb perforation is an extremely rare complication of colostomy. We report a case of sigmoid colostomy limb perforation due to diverticulitis occurring nine years after abdominoperineal resection for rectal cancer. An 83-year-old man presented with redness and pain around his stoma. He had undergone abdominoperineal resection nine years earlier, resulting in a permanent sigmoid colostomy. Initial CT suggested cellulitis, and antibiotics were administered, but his condition worsened. A repeat CT revealed an abscess, leading to incision and drainage, which confirmed stoma limb perforation. Segmental resection of the perforated bowel and same-site stoma reconstruction were performed. Histopathological examination indicated diverticulitis as the cause. The postoperative course was uneventful, and he was discharged on the 27th day. This case highlights the risk of late-onset stoma limb perforation due to diverticulitis. Contrast-enhanced imaging was essential for diagnosis, and long-term stoma care, including constipation prevention and regular imaging, is crucial for preventing similar complications.

## Introduction

A colostomy is not an uncommon surgical procedure performed for conditions such as rectal cancer, inflammatory bowel disease, and trauma. While it improves fecal diversion, it is associated with various complications, including peristomal dermatitis, parastomal hernia, stenosis, prolapse, and infections [1,2]. Among these, peristomal infections and abscess formation occur relatively frequently, whereas stoma limb perforation is extremely rare [3]. Although extremely rare, stoma limb perforation can lead to severe complications such as abdominal wall abscess and sepsis if not promptly recognized and treated. Its atypical presentation and delayed onset may contribute to diagnostic challenges, emphasizing the need for heightened clinical awareness.

Several factors have been suggested as potential causes of stoma limb perforation, including fecal impaction, parastomal hernia, ischemia, trauma, and inflammatory conditions such as diverticulitis [4,5]. Chronic intraluminal pressure and bowel wall fragility can lead to ischemic changes, increasing the risk of perforation over time [6].

Previous reports have indicated that stoma limb perforation can develop several years after colostomy creation, with some cases diagnosed more than a decade postoperatively [6]. However, such late-onset perforations are exceedingly rare. To our knowledge, only a few case reports have described similar presentations, including one that occurred 19 years after colostomy creation [6].

Here, we present a rare case of sigmoid colostomy limb perforation due to diverticulitis, occurring nine years after abdominoperineal resection for rectal cancer. This case highlights the importance of considering diverticulitis as a potential cause of late-onset stoma limb perforation and emphasizes the need for early diagnosis and appropriate management.

## Case presentation

An 83-year-old man was admitted to our hospital with complaints of redness and pain around his stoma. He had undergone abdominoperineal resection for rectal cancer nine years earlier, resulting in a permanent sigmoid colostomy. His medical history included hypertension, which was managed with oral medication, and no history of diabetes. He had been followed regularly in our outpatient clinic without evidence of cancer recurrence or postoperative complications until the current presentation. He had no history of stoma-related complications such as stenosis, parastomal hernia, or retraction. He also did not report symptoms of constipation or require regular use of laxatives.

**Figure 1:**
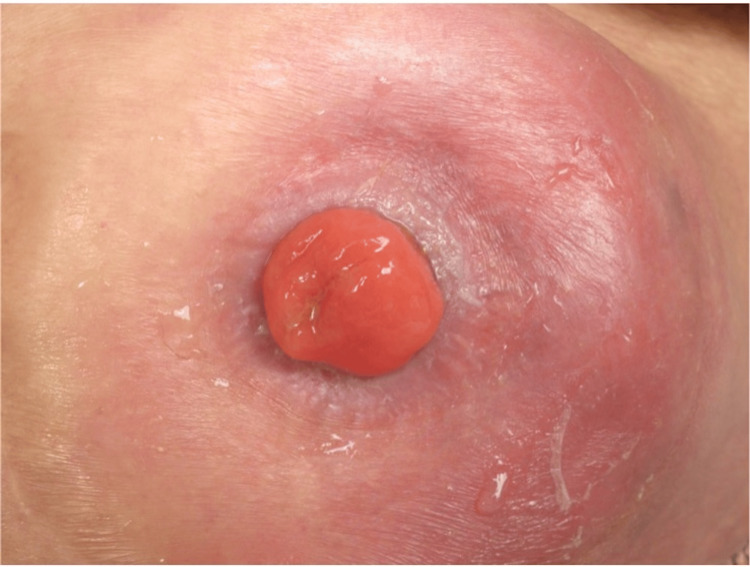
Localized erythema and swelling around the sigmoid colostomy. The stoma mucosa appeared healthy with no signs of necrosis, bleeding, or peritoneal irritation.

The patient's vital signs were as follows: blood pressure 110/58 mmHg, heart rate 62 beats/min, and body temperature 35.7°C. Physical examination revealed localized erythema, swelling, and tenderness around the left-sided sigmoid colostomy. The stoma mucosa appeared healthy without necrosis or bleeding, and there were no signs of peritoneal irritation (Figure 1). The stoma remained functional during this period. These findings suggested that overt ischemia was unlikely at the time of examination.

**Figure 2:**
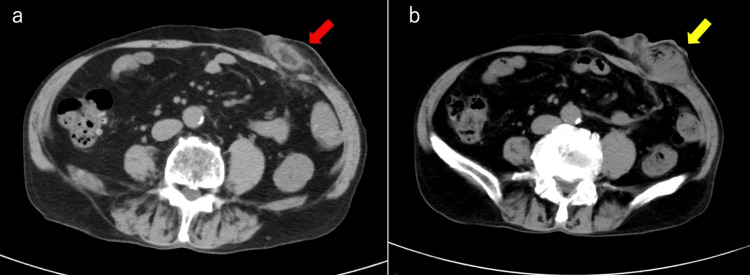
Abdominal CT findings. (a) Initial CT showing increased density in the subcutaneous fat surrounding the stoma (red arrow), consistent with cellulitis. (b) Repeat CT on the ninth day revealing gas and fluid collection (yellow arrow), indicating abscess formation.

Laboratory tests showed a mild elevation of white blood cell count and C-reactive protein (CRP), while blood glucose, electrolytes, and renal and liver function were within normal limits. Abdominal CT revealed increased density in the subcutaneous fat surrounding the stoma, consistent with cellulitis (Figure 2a). Oral cefcapene pivoxil (FMOX) 900 mg/day was started as empiric antibiotic therapy, resulting in initial symptomatic improvement. However, on the ninth day of hospitalization, swelling and pain at the stoma site worsened. A repeat abdominal CT demonstrated a marked increase in subcutaneous fat density with gas and fluid collection, indicating abscess formation (Figure 2b).

**Figure 3:**
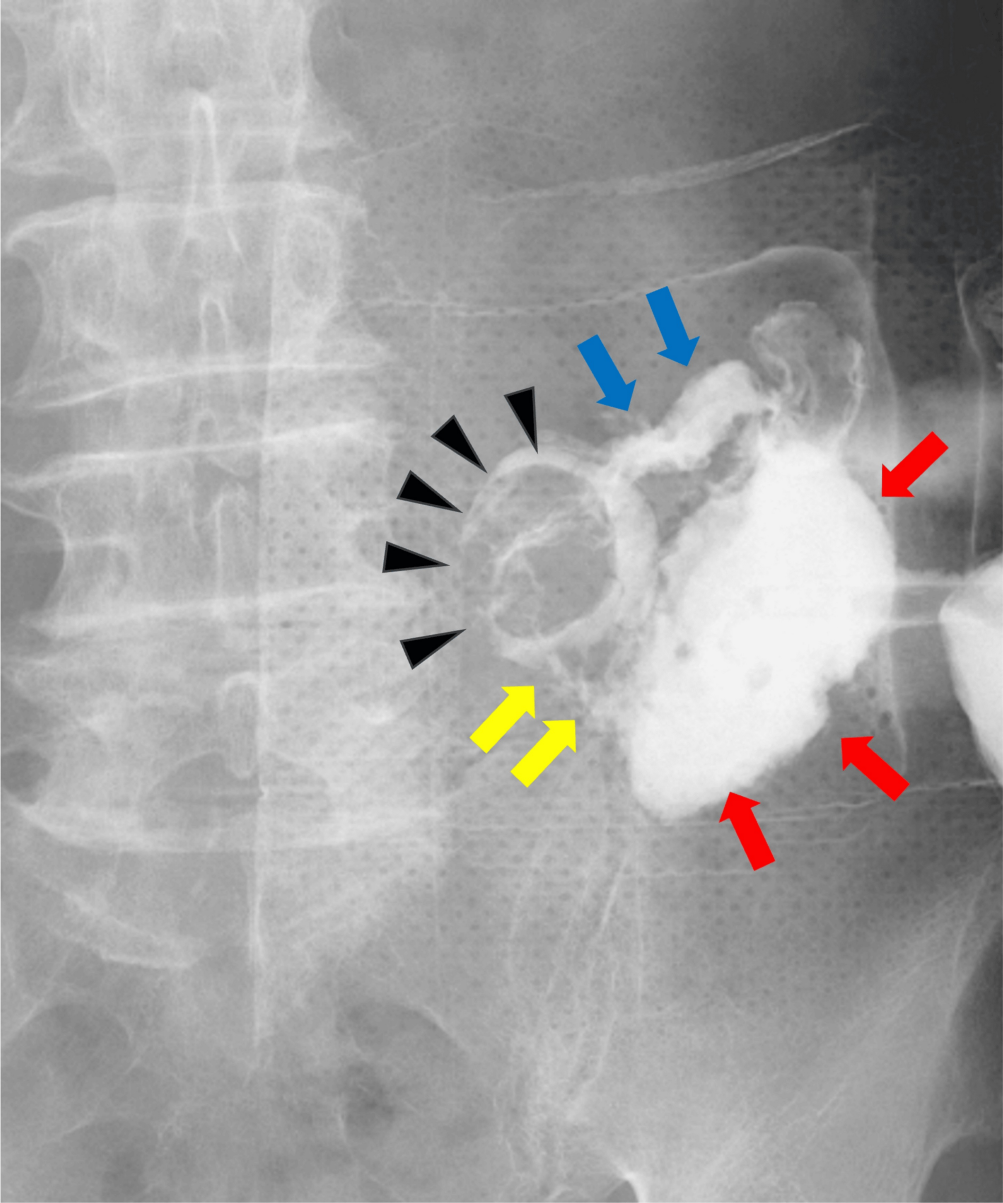
Contrast fluoroscopy from the drainage tube. The contrast study reveals an abscess cavity (red arrows) connected to the stoma (black arrowheads). The communication between the abscess cavity and the stoma is indicated by yellow arrows. The contrast-filled proximal bowel is marked by blue arrows.

Emergency incision and drainage were performed, and purulent fluid was evacuated from the abscess cavity. During the drainage procedure, contrast fluoroscopy via the drainage tube revealed leakage into the stoma limb, confirming the diagnosis of stoma limb perforation. Consequently, on the 16th hospital day, an exploratory laparotomy was performed (Figure 3).

**Figure 4:**
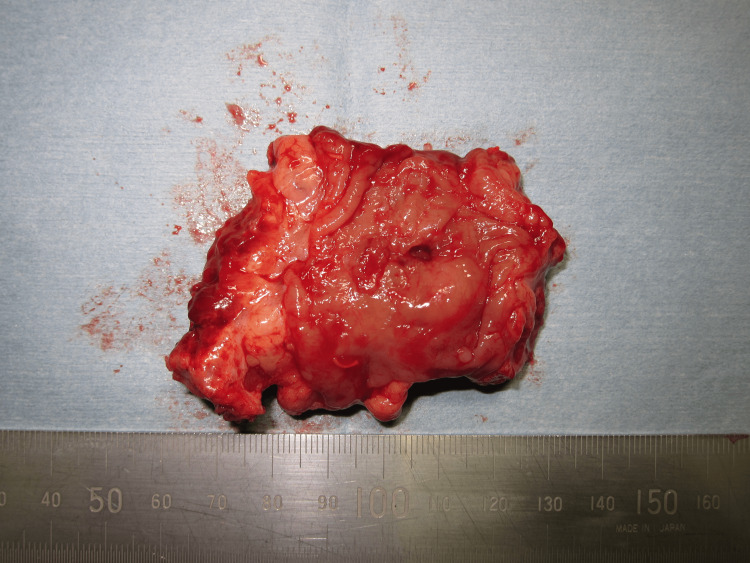
Macroscopic view of the resected specimen. A perforation is visible approximately 2 cm proximal to the stoma site. Severe inflammation and adhesions are observed around the perforation area.

Intraoperatively, a 5-mm perforation was identified approximately 2 cm proximal to the stoma. Severe inflammation and adhesions were observed around the perforation site. Segmental resection of the involved bowel, including the perforated segment, was performed (Figure 4). No signs of intraperitoneal contamination were detected. Therefore, no intra-abdominal drain was placed. The distal sigmoid colon was elevated and used to create a new colostomy at the same site. An open-type Nelaton drain was placed in the subcutaneous tissue around the colostomy site and was removed on postoperative day 3 due to favorable progress.

**Figure 5:**
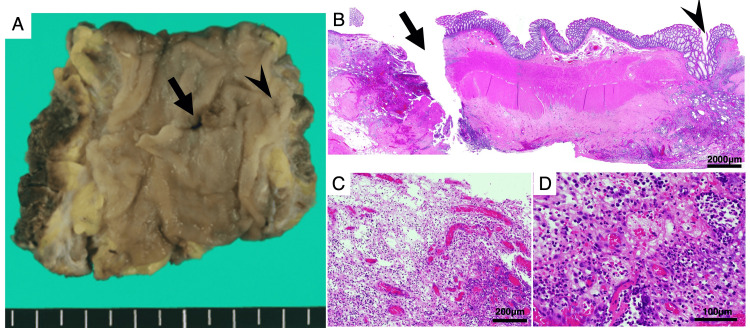
Histopathological examination of the resected specimen. Panel A shows the macroscopic appearance of a perforated diverticulum (arrow) and a nearby diverticulum (arrowhead) in the sigmoid colon after formalin fixation. Panel B shows the histopathological findings of the same site, revealing diverticulitis in the diverticulum indicated by the arrowhead. In Panel C, the perforated wall exhibits extensive infiltration of inflammatory cells and necrosis. Under higher magnification, granulation tissue with neutrophilic infiltration is observed (Panel D).

Histopathological examination of the resected specimen revealed a defect in the muscular layer consistent with a diverticulum. Severe infiltration of inflammatory cells and fibrosis were observed, confirming that the perforation resulted from diverticulitis (Figure 5).

The patient's postoperative course was uneventful, and he was discharged on the 27th postoperative day. The patient was followed up in the outpatient clinic. At five months after surgery, he developed a parastomal hernia, for which laparoscopic repair was successfully performed. He has since remained well without further complications.

## Discussion

Stoma limb perforation is a rare complication of colostomy. Common causes include fecal impaction, parastomal hernia, digital disimpaction trauma, and anti-vascular endothelial growth factor (anti-VEGF) therapy-associated bowel wall fragility [1,2,4,5,7]. Diverticulitis-induced perforation of the stoma limb, as seen in this case, is exceedingly rare, with only one other case reported by Watanabe et al. [5]. Additionally, this case occurred nine years postoperatively, making it the second longest interval reported after Nakamura et al.'s case (19 years) [6].

The mechanism of perforation is attributed to chronic intraluminal pressure from constipation, leading to diverticulitis and subsequent perforation. The passage of the stoma limb through the rectus abdominis muscle may cause angulation and luminal narrowing, predisposing the site to ischemia. Although such changes are typically associated with early postoperative complications, delayed onset may occur due to chronic low-grade inflammation, progressive tissue weakening, or mechanical stress over time.

Chronic bowel distension and microvascular compromise increase vulnerability to perforation, a mechanism consistent with this case [5]. Similar mechanisms have been reported in other cases, where increased intraluminal pressure due to fecal accumulation and impaired peristalsis contributed to bowel wall fragility [5,6]. Furthermore, chronic constipation and parastomal hernia have been implicated in elevated intraluminal pressure and subsequent perforation in stoma limbs [5,6].

Histopathological examination in our case revealed chronic inflammatory cell infiltration around the perforation site, without evidence of malignancy, supporting a non-neoplastic, pressure-related etiology. Similar mechanisms have been proposed in prior case reports, where persistent intraluminal pressure and ischemic changes contributed to delayed perforation [3,6].

While CT imaging plays a critical role in identifying complications such as abscesses and free gas, it may be limited in detecting early perforations or subtle ischemic changes, particularly in stoma limbs with chronic inflammation or fibrosis. In our case, the contrast-enhanced drainage study was instrumental in confirming the diagnosis by directly visualizing the communication between the abscess cavity and the colostomy limb. This technique should be considered in cases where standard imaging is inconclusive but clinical suspicion remains high.

In reported cases, the diagnosis of stoma limb perforation has been established primarily through abdominal CT scans, which effectively reveal localized abscess formation, increased fat stranding, and gas collections around the stoma site [1,8]. In this case, repeat CT scans demonstrated similar findings, suggesting stoma limb perforation.

However, a more definitive diagnosis was achieved by confirming the communication between the abscess cavity and the stoma limb through a contrast-enhanced drainage study. This approach allowed for a precise identification of the fistula, confirming the presence of stoma limb perforation. Utilizing contrast studies in conjunction with CT imaging provided a higher diagnostic accuracy, which was particularly valuable in this case where peritoneal irritation signs were absent.

Stoma limb perforations often present without peritoneal signs due to localized extraperitoneal involvement, as observed in this case [7,8]. This highlights the importance of maintaining a high index of suspicion and employing multiple diagnostic modalities to achieve an accurate diagnosis.

Surgical management in this case included segmental resection and reconstruction of the stoma at the same site. Same-site reconstruction has been reported to reduce operative trauma and facilitate postoperative wound management [3,9]. It also avoids complications associated with creating a new stoma site, especially in reoperative fields.

In contrast, reconstruction at a different site has been reported in cases with extensive inflammation or adhesions, necessitating a new location for stoma creation [5,7]. Additionally, Kim et al. reported cases of stercoral perforation where severe contamination and inflammation required stoma revision at a different site [10].

Enomoto et al. reported successful treatment with local drainage alone, suggesting that less invasive management may be effective for localized abscesses [11].

The postoperative course in this case was favorable, with discharge on the 27th day after surgery. Compared to other reported cases, this represents a relatively early discharge. Even when compared to Teruta [3], who performed same-site reconstruction and discharged on the 38th day, and Hoshi et al. [9], who performed reconstruction at a different site and discharged on the 64th day, this case showed a more rapid recovery.

In this case, the inflammation was localized to the abdominal wall, which likely contributed to the rapid recovery. Additionally, the absence of peritoneal irritation signs indicated a localized abscess, which may have been a key factor in the favorable postoperative outcome.

However, stoma limb perforation can lead to severe complications if the inflammation extends beyond the abdominal wall. In some cases, extraperitoneal abscesses can progress to sepsis and life-threatening conditions, especially when the diagnosis is delayed or if there is significant contamination [7]. In fact, Tamura et al. reported a case of stoma limb perforation leading to severe sepsis and requiring emergency surgery [7]. This highlights the importance of early recognition and prompt surgical intervention to prevent severe outcomes.

This case demonstrates that stoma limb perforation can occur even in long-term postoperative patients. The perforation occurred nine years after stoma creation, making it one of the longest intervals reported, second only to Nakamura et al., who reported a case 19 years postoperatively [6].

Since stoma limb perforation can occur even after a long postoperative period, careful monitoring is essential for patients with stoma creation. Maintaining a high index of suspicion for stoma-related complications and ensuring early diagnosis and prompt surgical intervention are crucial to prevent severe outcomes.

## Conclusions

Stoma limb perforation can develop many years after stoma creation, posing a significant risk of severe complications. Early recognition and timely surgical intervention are essential for improving patient outcomes. Long-term surveillance is essential for patients with long-standing colostomies. While routine imaging is not currently recommended in asymptomatic patients, timely evaluation based on symptoms, along with appropriate constipation management, may help prevent delayed diagnosis of rare but serious complications.

## References

[1] Sato G, Ikenaga M, Ueda M (2019). Two cases of stercoral perforation in the stoma limb early after colostomy. J Jpn Coll Surg.

[2] Iwata Y, Fukuta K, Suhara T, Furuta T, Miyazaki T (2017). A case of idiopathic colon perforation at the colostomy with parastomal hernia. J Jpn Coll Surg.

[3] Teruta S (2023). A case of stomal perforation treated by local surgery to avoid reforming the stoma by laparotomy. J Jpn Soc Stoma Contin Rehabil.

[4] Ozawa S, Kano K, Fujiuchi N, Sukigara M (2013). A spontaneous perforation of colostomy that occurred in self-care. J Jpn Coll Surg.

[5] Watanabe M, Sumi T, Udou R (2019). A case of diverticular perforation of sigmoid colostomy during FOLFIRI plus bevacizumab treatment [Article in Japanese]. Gan To Kagaku Ryoho.

[6] Nakamura S, Kuroda M, Takehara K, Ikeda E (2021). A case of idiopathic perforation in the stoma limb 19 years after colostomy. J Jpn Soc Coloproctol.

[7] Tamura T, Akiyama M, Okamoto K, Hirata K, Higure A, Nakayama Y, Yamaguchi K (2009). Abdominal wall abscess and sepsis induced by stercoral penetration of a sigmoid colostomy-report of a case. J Jpn Soc Abdom Emerg Med.

[8] Kataoka J, Nitta T, Ota M, Takashima Y, Imanishi M, Fujii K, Ishibashi T (2020). Successful multi-stage treatment of stoma limb perforation following Hartmann's operation report a case. Surg Case Rep.

[9] Hoshi Hoshi, Y Y, Igarashi T, Ishii S, Tamura H (2023). A case of subcutaneous colon perforation at the site of colostomy 4 years after Hartmann's operation. J Jpn Coll Surg.

[10] Kim YW, Kwon HJ, Kim IY (2013). Stercoral perforation of the colon in sigmoid colostomy patients: two case reports. Int J Surg Case Rep.

[11] Enomoto M, Sumi T, Tachibana S, Nagakawa Y, Tsuchida A (2021). A case of perforation of the stoma limb successfully treated by symptomatic treatment. J Jpn Soc Abdom Emerg Med.

